# NexGard^®^ Combo (esafoxolaner, eprinomectin, praziquantel), a new endectoparasiticide spot-on formulation for cats

**DOI:** 10.1051/parasite/2021013

**Published:** 2021-04-02

**Authors:** Frederic Beugnet

**Affiliations:** 1 Boehringer Ingelheim Animal Health 29 Av Tony Garnier 69007 Lyon France

## Introduction

The following articles in this special edition present the development studies regarding a new topical endectoparasiticide for cats containing three active substances: esafoxolaner (syn. (S)-afoxolaner), eprinomectin and praziquantel for cats with, or at risk of, mixed parasitic infestations with ectoparasites (fleas, ticks, and/or mites), gastrointestinal worms (gastrointestinal nematodes and cestodes), and cardiopulmonary worms (heartworm and/or lungworms).

This type of broad-spectrum combination is intended to protect cats against multiparasitism. Cats often have outdoor activities, including hunting prey, and are more at risk of multiple parasitic infestations than dogs. The sections below present the main parasites of cats and the specific case of multiparasitism.

## Epidemiological review of the main parasites of cats targeted by NexGard^®^ Combo

The treatment and prevention of external and internal parasites of pets is a recognized veterinary need. The major parasitological information and current recommendations from European parasitology experts are provided in guidelines from the European Scientific Counsel on Companion Animal Parasites [22, 24].

### Cat ectoparasites

#### Fleas

Fleas are blood-sucking insect parasites that occur on mammals and birds and are commonly found in cats, dogs and other small mammals housed in multi-pet households as companion animals.

The cat flea, *Ctenocephalides felis*, is one of the most frequent external parasites of companion animals worldwide and the most prevalent flea species in cats in Europe. Fleas can infest both dogs and cats all year round, but the infestation peak commonly occurs in late summer and autumn.

From a veterinary standpoint, fleas cause pruritus, alopecia, broken hairs, and allergic dermatitis in sensitized individuals, and anemia in heavily infested pets. Fleas also act as intermediate hosts or vectors for several pathogens (e.g.: *Dipylidium caninum*, *Rickettsia felis*, *Bartonella spp*., *Haemoplasma* spp.), which are all described in Europe.

The prevalence of fleas found on cats is highly variable based on the epidemiological surveys: 14% in Germany [3], between 21% and 56% in the United Kingdom (UK) [4], and up to 97% in Greece [32].

#### Ticks

In Europe, the ticks found on cats are members of the Ixodidae and include species within the main genera *Ixodes* and *Rhipicephalus,* and more rarely *Dermacentor*. In the United States, *Amblyomma* spp. ticks also commonly infest cats.

The main European tick species in cats is *Ixodes ricinus,* which is widely distributed, except in southern Europe. *Rhipicephalus* is primarily a Mediterranean tick of southern Europe, while *Dermacentor* is found throughout Europe with patchy distribution.

Tick infestation is highly seasonal. In the UK and Central Europe, there are typically two peaks, one from March to June and a second from August to November. In more southern climates, the prevalence increases in spring and summer, but the ticks may feed all year round if temperatures are conducive [24].

The direct pathogenic effect of ticks is linked to their blood meal, which may lead to anemia in cases of heavy infestations. There might also be local inflammatory reactions at the site of the bite. Generally, ticks are of most importance as vectors of pathogens affecting both companion animals and humans [23].

#### Otodectes cynotis

Ear mites, *Otodectes cynotis*, are a cause of aural irritation and discomfort in cats, dogs and ferrets. They can occur in any age group of cats but are more common in kittens. They spend their entire lifecycle on the host, with transfer from animal to animal occurring through close contacts [12]. Ear mites are found all around the world. In a survey performed in domestic owned cats in Europe, *O. cynotis* was the most frequently identified species (17.4%) [5]. In another survey conducted in Italy, *O. cynotis* was identified as the primary cause of external otitis in 53.3% of 1087 stray cats examined [42]. Ear mites may be tolerated without clinical signs in some animals, but can cause pruritus with ear scratching or rubbing, ear pinnae hematoma, and self-inflicted trauma.

### Cat endoparasites

#### Gastrointestinal nematodes

Infections with gastrointestinal nematodes (ascarids *Toxocara cati* and *Toxascaris leonina*, and hookworms, *Ancylostoma* spp. and *Uncinaria stenocephala*) occur by ingestion of larvated eggs or free larvae from the environment, for ascarids and hookworms, respectively. Other modes of infections include larvae ingestion through the milk in young kittens, or the ingestion of paratenic hosts, such as small rodents in hunting cats.

*Toxocara cati* is the most common digestive parasite of the cat worldwide. Except in young animals or when there is a massive infestation, *T. cati* usually induces limited digestive clinical signs (e.g. vomiting, diarrhea). The pulmonary migration of L4 larvae also causes pneumonia with coughing [49].

*Toxocara cati* has zoonotic potential. Human infections are mainly asymptomatic, but fever, persistent eosinophilia, and hepatomegaly (sometimes with pulmonary involvement) may occur, resulting in a condition known as visceral larva migrans [14].

*Toxascaris leonina* infection is rare and mainly occurs in rural environments. Paratenic hosts play a major role as a source of the parasite.

Cats can become infected with several hookworm species, including *Ancylostoma tubaeforme,* the main hookworm species observed in cats, and *Ancylostoma braziliense* or *Ancylostoma ceylanicum* in tropical countries. All species are blood-suckers, but they usually cause only mild digestive disorders and occasional diarrhea in cats. They may also induce hypoalbuminemia and anemia. The main importance of *A. braziliense* and *A. ceylanicum* is related to their zoonotic potential. *A. braziliense* can cause cutaneous larva migrans or “creeping eruption” in humans [17], while *A. ceylanicum* develops into the adult stage and is the second most common hookworm species observed in humans in Asia. People become infected when the zoonotic hookworm infective larvae penetrate unprotected skin.

*Toxocara cati* and *Toxascaris leonina* are found in cats all over Europe. *T. cati* has been shown to have a high prevalence, ranging between 11% and 60% in various coprological and postmortem surveys [2, 56].

The hookworm *Ancylostoma tubaeforme* is endemic in domestic cats throughout the world. In Europe, it is most frequently observed in Italy, Austria, Belgium and Spain, with prevalence ranging from 1% to over 30% depending on the surveys [10, 50].

#### Gastrointestinal cestodes

Infection by gastrointestinal cestodes is common in cats. The route of infestation by the various tapeworms differs: the intermediate host of *Dipylidium caninum* is the flea, while that of *Taenia taeniaeformis* and *Echinococcus multilocularis* is small rodents hunted by the cat. Recent findings have demonstrated that cats are infected by a different genotype of *Dipylidium* than dogs. These two genotypes, i.e. canine and feline, are also clearly distinguished in the fleas collected on dogs or on cats, the dog genotype being the only one observed in *Ctenocephalides canis*. The genetic and biological differences, i.e. absence of hybrids and difficulty to infect cats with the dog genotypes and vice versa, have led authors to suggest that these are two distinct species of *Dipylidium* [7, 33].

Adult tapeworm infections seldom cause severe clinical signs in the cat, unless large numbers of tapeworms are present [8].

Cestode infections may represent a risk to human health [13]. *Dipylidium caninum* can exceptionally cause zoonotic infection in children following the accidental ingestion of fleas carrying the larval stage of the tapeworm. Although human infection is mostly nonpathogenic, it may lead to anorexia and weight loss in infected children [38].

A far more important zoonotic agent is *Echinococcus multilocularis* which causes alveolar echinococcosis in humans. Human infections are fortunately rare, but can be fatal without extensive chemotherapy and/or surgery. Cats are generally thought to be less suitable hosts for *E. multilocularis* than canids, with a reduced rate of worm development [53]. However, cases of heavily infected cats are known to occur [18].

Cat infection with *Dipylidium caninum* and *Taenia taeniaeformis* is extremely common in Europe. As an illustration, an Austrian survey found that 33% of cats were infected with *T. taeniaeformis* [28], as were 20–28% of cats sampled in Belgium [56].

One difficult aspect of teniosis is its diagnosis. The sensitivity of coproscopy remains very low. *Dipylidium* proglottids usually exit the rectum mechanically, with no link to defecation. This enables proglottids and eggs present in the cat environment, pet bedding, and sofas to be ingested by flea larvae, which are not on feces. Recent studies comparing necropsy and coproscopy in feral cats have indicated sensitivity of around 10% for coproscopy [51]. Regarding infection by *Taenia taeniaeformis*, sensitivity is higher, around 30–40%, as some proglottids disrupt and lay eggs in the large intestine [51].

Another way of assessing the epidemiological status of *Dipylidium caninum* is the check fleas for flea larvae through PCR techniques. On average, 4% of fleas are found to be infected in Europe [6].

*Echinococcus multilocularis,* which is primarily a parasite of foxes, occurs in large areas of Europe and is endemic in countries of Central and Eastern Europe [22]. Several studies have reported *E. multilocularis* infections in domestic cats. As an example, 3.7% of cats were found to be infected in a survey in the east of France [43], and they are able to excrete proglottids and eggs [40].

*Joyeuxiella* species, such as *Joyeuxiella pasqualei* and *Joyeuxiella fuhrmanni*, are common cestodes of cats from the Middle East, Southern Asia, and Africa [8]. Data on diagnoses/findings of infections with *Joyeuxiella* cestodes are rare in Central European countries. However, cases of *Joyeuxiella* infestations are reported in other European countries, especially in South–Eastern European countries and those bordering the Mediterranean Sea [10, 27, 30, 36, 37, 41, 44, 45, 46, 48, 57].

#### Cardiopulmonary worms

##### Heartworm

*Dirofilaria immitis*, the canine and feline heartworm, is a nematode transmitted by mosquitoes, living in the arteries of the lungs, and occasionally in the right side of the heart of dogs and cats.

Heartworm infection is an unpredictable disease in cats. Most cats show no clinical signs for a long time after infection. These cats may undergo spontaneous self-cure due to the natural death of parasites or they may suddenly show a dramatic acute syndrome with respiratory signs. Sudden death in apparently healthy cats is not an infrequent consequence of infection. In most cases, the onset of clinical signs seems to be related to the natural death of parasites or to the arrival of pre-adult heartworms in the pulmonary arteries. Ectopic localizations in the brain, eyes, testis or aorta occur rarely, and are more frequent in cats than in dogs [22].

*Dirofilaria immitis* is endemic in the Mediterranean region, including many southern and south-eastern European countries. The prevalence rate of naturally acquired infections in cats is between 5% and 20% that for dogs in the same geographical area [34]. Various studies have shown that in endemic areas in Italy, up to 27% of cats may be infected. The infestation is seasonal, linked to the presence of the vectors (mosquitoes), generally active from April to October.

It should be noted that mosquito density and the rate of *Dirofilaria* maturation to infective third-stage larvae in the mosquito vector depend mainly on environmental temperature and humidity. A rise in average temperatures as well as the emergence of a new vector, *Aedes albopictus* in Europe, has tended to extend the risk areas and the risk season for infection, and this contributes to an increased prevalence rate [54].

##### Lungworms

*Aelurostrongylus abstrusus* infects the lung parenchyma, the terminal respiratory bronchioles, and alveolar ducts of lungs in cats and has an indirect cycle [8]. Feline aelurostrongylosis may be asymptomatic, depending on worm burden, age and immune response of the infected animal. However, the disease is generally characterized by respiratory signs that may be severe and may result in death in the young, debilitated, and/or immunosuppressed cat [55]. Complications may include interstitial emphysema, pulmonary edema, and secondary bacterial pneumonia.

*Troglostrongylus brevior* is a respiratory nematode with an indirect lifecycle like *Aelurostrongylus abstrusus*. Clinical signs in cats infected with *Troglostrongylus brevior* can be severe, and may include cough, dyspnea, and nasal discharge, and may lead to fatal respiratory failure. Kittens and young cats seem to be more likely to develop clinical signs than adult animals [9, 20].

Recent reports support the presence of *A. abstrusus* everywhere in Europe. There are endemic areas in Europe, like Portugal and Italy where the prevalence of this lungworm is about 20% [55]. The overall prevalence of *T. brevior* in domestic cats within Europe is low in general and cases are mostly found in countries bordering the Mediterranean Sea [19, 21, 26, 29, 31, 52].

#### Vesical worms

*Capillaria plica* is a nematode that infects the urinary bladder of carnivores, and occurs worldwide, mostly in wild animals [30]. This infection is rarely reported in domestic carnivores, but the available data indicate reports in cats, which have more frequent contact with the external environment. *Capillaria plica* infection can induce cystitis, and the infection is often diagnosed accidentally, when examining urine sediment. Consequently, this infection may also cause pollakiuria, dysuria, and hematuria [47].

### Co-infections

The parasites targeted by NexGard^®^ Combo (fleas, ticks, ear mites, nematodes and cestodes) are commonly found in cats in Europe and can be present simultaneously in the same animal. This explains why many endectoparasiticide medicinal products have been developed for pets in recent years (e.g. Advocate [Bayer AH], Broadline [Boehringer Ingelheim], Bravecto Plus [MSD], and Stronghold Plus [Zoetis]).

The risk of cats being infected by any type of gastrointestinal helminth seems to be high in Europe. Recent European studies revealed the occurrence of concurrent nematode and cestode infection in 5%–14% of client-owned cats [1, 11]. Even higher prevalences are reported in stray cats.

In addition, an epidemiological survey was conducted in 2014 and highlighted for the first time that multiparasitism is frequent in European owned cat populations. This survey demonstrated that more than half of the owned cat population carries at least one parasite at a given time, with a high level of co-infestations. Overall, 50.7% of cats were found to be positive for at least one internal or one external parasite species. Co-infection with endoparasites and ectoparasites was found in 14% of the cats, and 11.9% harbored both ectoparasites and gastro-intestinal helminths [5, 25]. The same findings regarding cat parasites are made for other countries or regions of the world like the United States [39] and Asia [16]. Given the zoonotic considerations and the clinical importance, it is strongly advisable to promote effective and regular parasite control in cats for both ectoparasites and endoparasites.

Due to the modalities of infestation (e.g. ingestion of eggs or larvae, preying small mammals), cats that go outdoors are obviously at greater risk of exposure to both nematode and cestode infections. Interestingly, there is also evidence that even cats without outdoor access are at risk of helminth infections: in an evaluation on the helminth prevalence in cats in Germany and France, 20% of cats that were positive for helminth infection did not have outdoor access [15]. Other risk factors should be considered such as age (kittens and geriatric animals are at greater risk than healthy adults for *T. cati* infection), environment (co-housed cats in catteries or individual households, and multi-pet households), health status of the animals including ectoparasite infestation, or geographic location and travel that may make exposure to certain parasites more likely.

### Conclusion

The parasites targeted by broad spectrum endectoparasiticides (fleas, ticks, mites, gastrointestinal nematodes, cestodes, heartworms, and lungworms) are quite commonly found in cats, and can occur simultaneously as concurrent infestations (ecto- and endoparasitism). Therefore, there is an interest for veterinarians and cat owners to provide a product combining such treatment claims.

## NexGard^®^ Combo for cats

In the following articles, the major development studies regarding the pharmacokinetics, and safety and efficacy of the association of the three active ingredients (esafoxolaner, eprinomectin and praziquantel) are described.

It was first demonstrated that the three active substances do not interact with one another. Thereafter, the pharmacokinetic profile supported the deworming curative effect and sustained insecticidal-acaricidal activity for at least one month.

The required frequency of treatment of cats with this broad-spectrum combination will vary, as it depends on parasitological and epidemiological considerations. Certain factors may dictate more intensive monitoring and/or treatments, while others may suggest less frequent use. Regarding identification of the parasitic risk, the veterinarian is paramount for the prescription recommendation and the frequency of treatment. He/she should consider the cat’s individual risk factors such as age, health status, housing, lifestyle, family environment (zoonotic risk), and geographical location or travel. It is important to be aware of the limited understanding of owners regarding the need to protect cats against several types of parasites, and not only fleas [35].

Thus, the veterinary prescription must be based on a comprehensive reasoned risk assessment of each individual cat.
